# Modeling and evaluation of causal factors in emergency responses to fire accidents involving oil storage system

**DOI:** 10.1038/s41598-021-97785-4

**Published:** 2021-09-24

**Authors:** Changfeng Yuan, Yulong Zhang, Jiahui Wang, Yating Tong

**Affiliations:** 1 School of Maritime Economics and Management, Dalian Maritime University, Dalian, 116026 China; 2 718 Research Institute of CSIC, Handan, 056027 China

**Keywords:** Environmental sciences, Energy science and technology, Engineering

## Abstract

According to the statistics of 160 typical fire and explosion accidents in oil storage areas at home and abroad nearly 50 years, 122 of them occurred the secondary accidents in the emergency responses. Based on 122 accident cases, 21 causal factors leading to secondary accidents are summarized. In order to quantify the influencing degree of these causal factors on the accident consequences, a multiple linear regression model was established between them. In the modeling process, these factors are decomposed into the criterion layer, variable layer, and bottom layer. The improved analytic hierarchy process (IAHP) was used to establish the relationship between the bottom factors and variable factors, and the regression analysis method was used to establish the relational model between variable layer and criterion layer. For 122 cases of the secondary accidents, this study took the year as a statistical dimension, and obtained 40 groups of sample data. The first 34 groups of sample data were used to build the causal factors model, and the last 6 groups of sample data were tested the generalization ability of the model by using the established regression model combined with grey prediction model. The results show that the prediction ability of the established model was better than that of the grey prediction model alone. Moreover, the relative contribution and change trend of the causal factors were evaluated using the mutation progression method, and corresponding preventive countermeasures were proposed. It was found that human professional skills, knowledge and literacy, environmental issues, and firefighting facilities are the main influencing factors that lead to the secondary accidents. These three kinds of factors show a gradual improvement trend, and the existing prevention measures should be maintained and further improved. The problem of inherent objects or equipment factors has not been effectively improved and has a worsening trend, which is the focus of prevention in the future, and the prevention and control efforts need to be moderately increased. The research results have important guiding significance for understanding the quantitative influences of causal factors on the accident consequences, improving emergency response capabilities, reducing accident losses, and avoiding secondary accidents.

## Introduction

Safe oil storage system is important to ensure the safety of people's livelihoods and the healthy development of the economy. Once a fire or explosion accident occurs during oil storage system, it may cause serious consequences such as casualties, economic losses, and environmental pollution. However, much can be learned from real-world data. For example, secondary accidents caused by improper emergency responses have sometimes occurred, which have increased the casualties, expanded the accident situation, and increased environmental pollution. According to the statistics, just in China in 2017^1^, there were more than eight accidents with increased casualties (41 people were killed and 16 injured) as a result of inappropriate emergency disposal in the petrochemical industry. It can be seen that there are potential causal factors in the emergency responses to accidents and post-reconstruction process. These causal factors and their interactions are the keys to secondary accidents and serious consequences. Determining these potential causal factors and establishing the relationships between them and the consequences of an accident could quantitatively determine their effects on the consequences of the accident, which could provide a scientific basis for more targeted active protection beforehand.

At present, analyses of the causal factors for fire accidents in oil storage systems are mainly based on the factors influencing the initial accident, and the analysis methods mainly adopt qualitative or quantitative methods. For example, in a qualitative analysis method, the fishbone diagram analysis method is used to determine the factors influencing oil and static electricity accidents, and the analytic hierarchy process (AHP) is used to determine the importance of each influencing factor^2^. Feng et al.^3^ used the interpretative structural modeling (ISM) theory to establish an explanatory structure model for the causes of third-party damage to oil and gas pipelines, constructed a six-layer hierarchical structure integrated system, and calculated the weights of the factors at each level of the hierarchical system using the optimal order comparison method to find the key factors. Huang et al.^4^ took the Qingdao oil and gas pipeline leakage and explosion accident as an example and used the AHP to analyze the human factors influencing the oil and gas pipeline leakage and explosion and determine the weight of each influencing factor. Qualitative analysis methods can better describe the relationships between the factors and the relative importance of each factor. However because of human subjectivity, it is difficult to accurately determine the quantitative influence and relative contribution of each factor to the accident.

In the quantitative analysis methods, on one hand, the causal factors are analyzed using some quantitative methods, such as, the fault tree analysis (FTA) method, a Bayesian network (BN), or a combination of the two often used. For example, FTA^5–8^ is used to quantitatively analyze the factors influencing fire and explosion accidents involving oil storage tanks, and to calculate and sort the structural importance of each factor. The BN method^9,10^ was used to analyze the probabilities of the causal factors in a natural gas explosion accident. Some scholars^11^ have used the FTA and BN methods to analyze the causal factors of leakage in submarine oil and gas pipelines. Ma et al.^12^ analyzed the causal factors and their degree of influence for fire and explosion accidents at a gas station based on fault trees and improved Bayesian network methods.

On the other hand, quantitative analysis is carried out by establishing a correlation between the causal factors and the consequences of the accident. Cui et al.^13^ introduced the fuzzy bow-tie model in quantitative risk analysis to quantitatively analyze the possibility and consequences of oil spill accidents, and then proposed specific risk prevention and control measures. Yu et al.^14^ applied the protection layer analysis method, established an independent protection layer model, found the root cause of an accident, used the protection layer model to analyze the occurrence probability of the consequence event, and proposed a complete semi-quantitative analysis method for urban gas pipeline failure and risk assessment. Zhao et al.^15^ used PHAST software to quantitatively analyze the influences of factors such as the wind speed and blowout pressure on the consequences of an accident. Shang et al.^16^ analyzed the leakage and diffusion law for urban LPG pipelines and their influencing factors, and used the RNGk-ε model to analyze the impact of the environmental wind speed, obstacles, and urban topographical conditions on the consequences of LPG leakage accidents using LPG pipelines in a certain city.

In summary, the current research on the causal factors of fire accidents involving oil storage system focuses primarily on the initial accident. It mainly uses qualitative or quantitative analysis methods, or existing risk assessment models, to analyze the effects of the causal factors on the consequences of accidents. However, from the current literature, there is no public report on the establishment of a quantitative impact model from the perspective of the relationship between the causal factors of a secondary accident and the consequences of the accident. Therefore, based on a statistical analysis of 160 typical fire and explosion accidents in oil storage areas publicly reported at home and abroad over the past 50 years, this paper summarizes 21 causal factors leading to secondary accidents and their frequencies. A multiple linear regression model between the causal factors of a secondary accident and the accident consequences was established through the multi-level decomposition of the causal factors and the establishment of the correlation between layers. Moreover, the relative contribution of each causal factor was evaluated, and the main influencing factors affecting the accident consequences were determined by analyzing the change trend. This made it possible to propose corresponding proactive preventive measures.

## Statistical analyses of causal factors

According to the statistics for 160 typical fire and explosion accidents in oil storage areas at home and abroad from 1971 to 2020^17–19^, 122 (including 75 in China and 47 in foreign countries) secondary accidents occurred. The 160 accident cases collected in this paper are the statistical data of fire and explosion accidents occurred in the oil reservoir area and involving the oil storage tank and its accessories (such as oil pipeline, discharge pipe, fire cooling sprinkler system, fire foam system, etc.), which are recorded in public reports and literature. Based on 122 accident cases, the classified risk source identification method^20^ is adopted to identify the major influencing factors that occur frequently in emergency responses. From four aspects: humans, materials, environment, and management^21^, 21 main causal factors leading to secondary accidents are obtained. The labels for these causal factors and their frequencies are listed in Table 1.

**Table 1:** Statistics on causal factors and frequencies.

Causal factors	Frequency	Causal factors	Frequency
Illegal operation	3	Not setting firefighting facility	5
Not handling in time	6	Firefighting facility failure	25
Misjudgment	2	Fire water system failure	7
Management mechanism	79	Unreasonable setting of fire dike	3
Safety laws and regulations	59	Drain valve failure	2
Tank broken	54	Small fire separation	6
Valve broken	13	Reburning or reblasting of high-temperature oil	18
Flange broken	4	Oil leakage and spillage	45
Oil pipelines broken and leakage	10	Blast wave and radiant heat	63
Floating plate damage	10	Weather factor	14
Power-supply system and water system broken	8		

Frequency refers to the occurrence times of each causal factor in the 122 accident cases.

## Modeling and analysis of causal factors

### Modeling idea of causal factors model

As can be seen from Table 1, there are 21 main causal factors that lead to secondary accidents in the emergency processes. Taking these factors directly as variables would undoubtedly be the best way to reflect the corresponding relationships between the causal factors and the accident consequences. However, because of the large number of causal factors, this would lead to too many independent variables in the model, and the modeling accuracy would be difficult to guarantee. Therefore, based on the layered construction principle (namely, layer-by-layer modeling principle) of the model, these causal factors are decomposed by multiple layers, and the corresponding relationship between the bottom layer and the dependent variables is indirectly found by establishing the direct relationship between factors at adjacent layers. In this study, the causal factors were decomposed into three levels, which were called the "criterion layer (first level)," "variable layer (second level)," and "bottom layer (third level)" from top to bottom. Among these, the criterion layer included human, material, and environmental factors. The management factors previously counted were incorporated into the human factors based on the three types of hazard sources^22^. Because the implementation and operation of management factors are inseparable from human behavior, they can be controlled and implemented by human factors. Moreover, a large number of accident statistics show that human factors (including human operation, organization management, plan design, decision-making errors) are the most important causes of accidents. The variable layer factors were subdivisions of the criterion layer factors. Among these, the material factors were divided into inherent objects or equipment (such as storage tanks and oil pipelines) and firefighting facilities based on the different equipment failures. The bottom layer included 21 causal factors derived from the statistics. Table 2 shows the hierarchical decomposition of the causal factors in the emergency responses to fire accidents involving oil storage system.

**Table 2:** Level decomposition table of causal factors in emergency responses.

Total set	Criterion layer	Variable layer	Bottom layer
Causal factors leading to secondary accidents	Human factors	Human professional skills, knowledge, and literacy (F_1_)	Illegal operation (X_11_)
			Not handling in time (X_12_)
			Misjudgment (X_13_)
			Management mechanism (X_14_)
			Safety laws and regulations (X_15_)
	Material factors	Inherent object or equipment (F_2_)	Tank broken (X_21_)
			Valve broken (X_22_)
			Flange broken (X_23_)
			Oil pipelines broken and leakage (X_24_)
			Floating plate damage (X_25_)
			Power-supply system and water system broken (X_26_)
		Firefighting facilities (F_3_)	Not setting firefighting facility (X_31_)
			Firefighting facility failure (X_32_)
			Fire water system failure (X_33_)
			Unreasonable setting of fire dike (X_34_)
			Drain valve failure (X_35_)
			Small fire separation (X_36_)
	Environmental factors	Environmental issues (F_4_)	Reburning or reblasting of high-temperature oil (X_41_)
			Oil leakage and spillage (X_42_)
			Blast wave and radiant heat (X_43_)
			Weather factor (X_44_)

### Modeling method of causal factors model

#### Preprocessing of input and output variables in causal factors model

The relationships between the consequences of an accident and the causal factors have significant randomness. The construction process for the causal factors model needs to decrease the randomness of the accident system to obtain the overall characteristics of a certain type of accident, so that it has more reference significance. Therefore, time is required as a statistical dimension when modeling the causal factors obtained from multiple case accident statistics. The output of the model is the accumulation of the severity values of all the major accidents (the severity of the accident is reflected in the number of deaths) within a certain period of time t, and the input variable of the model is the overall characteristic value of the accident factors within time t.

Based on this, for 122 statistical accident cases, this study took the year as a statistical dimension, and obtained 40 groups of sample data. The last six groups of sample data were reserved for prediction testing, and the first 34 groups of sample data were used to build the causal factors model, which was established using the regression analysis method. The form of this model is shown in Eq. (1):

1 $${\mathrm{y}}\left({\mathrm{j}}\right)={a}_{0}+\sum_{i=1}^{n}{a}_{i}{f}_{i}(j)$$

where ${\mathrm{y}}\left({\mathrm{j}}\right)$ is the cumulative value of the accident consequences of all the accidents in the ${j}$th group, ${f}_{i}(j)$ is the eigenvalue of the ${i}$th influencing factor in the ${j}$th group, ${a}_{i}$ is the characteristic constant of the ${i}$th influencing factor, and ${a}_{0}$ is the characteristic constant.

#### Determination of eigenvalues in variable layer factors

According to Eq. (1), in order to establish the causal factors model, it is necessary to determine the eigenvalues of the variable layer factors. According to the layer-by-layer modeling principle, there must be a certain linear relationship between the eigenvalues at the variable layer factors and those at the bottom layer factors, and the relationship between them could be obtained by Eq. (2).

2 $${f}_{i}\left(j\right)=\sum_{k=1}^{p}{\beta }_{ik}{x}_{ik}(j)$$

where ${\beta }_{ik}$ is the weight of bottom layer factor ${x}_{ik}(j)$, ${f}_{i}(j)$ is the eigenvalue of the ${i}$th variable layer factor in the ${j}$th group, and ${x}_{ik}(j)$ is the cumulative eigenvalue of the ${k}$th bottom layer factor under the ${i}$th variable layer factor in the ${j}$th group.

The eigenvalues of the bottom layer factors were represented by binary numbers, where "0" indicated that the causal factor did not appear in the accident, and "1" indicated that the causal factor did appear in the accident. Among the 40 pieces of sample data, the cumulative eigenvalues of the bottom layer factors (that is, the values of ${x}_{ik}(j)$) are listed in Table 3.

**Table 3:** Cumulative eigenvalues of bottom causal factors.

Seq	X_11_	X_12_	X_13_	X_14_	X_15_	X_21_	X_22_	X_23_	X_24_	X_25_	X_26_	X_31_	X_32_	X_33_	X_34_	X_35_	X_36_	X_41_	X_42_	X_43_	X_44_
1	0	0	0	1	0	0	0	0	1	0	0	0	0	0	0	0	0	0	1	1	0
2	0	0	0	1	0	1	0	0	0	0	0	0	0	0	0	0	0	1	1	1	0
3	0	0	1	1	1	0	0	0	0	0	0	0	1	0	0	0	0	0	1	0	0
4	0	0	0	1	0	0	0	0	0	0	0	0	0	0	0	0	0	0	0	1	0
5	0	0	0	2	2	1	0	0	0	0	0	0	1	0	1	0	0	1	1	1	1
6	1	0	0	1	1	0	0	0	0	0	0	0	0	0	0	0	0	1	1	1	1
7	0	0	0	1	1	0	0	1	0	1	0	1	0	0	0	0	0	0	0	0	0
8	0	0	0	2	2	3	0	0	0	0	0	1	0	1	0	0	0	1	1	3	1
9	1	0	0	2	2	1	0	0	1	0	0	0	1	0	0	0	0	0	0	1	0
10	0	0	0	1	1	0	1	0	0	0	0	0	0	0	0	0	0	1	1	1	1
11	0	0	0	1	1	2	1	0	0	0	0	0	0	0	0	1	0	0	1	2	0
12	0	0	0	2	2	0	0	0	1	1	0	0	2	0	1	0	0	0	1	2	1
13	0	0	0	3	3	1	0	0	0	0	1	0	1	0	0	0	1	1	1	2	2
14	0	1	0	2	2	1	0	0	0	0	0	0	1	0	0	0	0	2	2	0	0
15	0	0	0	2	1	1	0	0	0	0	0	0	1	0	0	0	2	0	1	2	0
16	0	0	0	3	1	1	0	0	0	2	0	0	1	0	0	0	0	1	0	1	2
17	0	1	0	2	1	1	0	0	1	1	0	0	0	1	0	0	0	0	2	2	0
18	0	0	0	1	1	1	0	0	0	0	0	0	0	0	0	0	0	0	0	0	0
19	0	0	1	1	1	1	1	0	0	0	0	0	0	0	0	0	1	1	0	1	1
20	0	0	0	2	2	1	1	0	0	0	0	0	0	0	1	0	0	0	1	1	0
21	0	0	0	3	3	1	0	0	1	0	0	0	2	2	0	0	0	1	2	1	0
22	0	0	0	3	3	2	0	0	0	1	0	0	2	0	0	0	0	1	2	0	0
23	0	1	0	2	1	1	0	1	0	1	0	0	0	0	0	0	0	0	0	2	1
24	0	0	0	1	0	1	0	0	0	0	0	0	0	0	0	0	0	0	0	0	0
25	0	1	0	2	1	0	0	0	0	0	0	0	0	0	0	0	0	1	0	2	0
26	0	0	0	1	1	2	0	0	0	0	0	0	0	0	0	0	0	0	0	2	0
27	0	0	0	2	2	3	1	1	0	0	1	1	1	1	0	0	1	1	2	2	0
28	0	0	0	1	0	0	0	0	0	0	0	0	0	0	0	0	0	0	0	1	0
29	0	1	0	2	2	0	1	0	0	0	0	0	1	0	0	1	1	0	2	1	0
30	0	0	0	5	4	3	1	0	0	1	1	1	3	1	0	0	0	1	1	5	1
31	0	0	0	2	2	0	1	0	1	0	1	0	1	0	0	0	0	1	2	1	0
32	0	1	0	4	2	0	0	0	0	0	1	0	0	0	0	0	0	0	2	1	0
33	0	0	0	7	6	2	2	0	0	0	0	0	1	1	0	0	0	2	6	4	1
34	0	0	0	5	2	2	0	0	0	1	1	1	2	0	0	0	0	0	1	4	0
35	0	0	0	1	1	5	1	0	0	1	0	0	1	0	0	0	0	0	2	5	1
36	0	0	0	1	0	2	2	0	0	0	0	0	0	0	0	0	0	0	0	2	0
37	0	0	0	2	2	1	0	0	2	0	2	0	1	0	0	0	0	0	3	4	0
38	0	0	0	0	0	0	0	0	0	0	0	0	0	0	0	0	0	0	2	1	0
39	0	0	0	1	0	0	0	0	1	0	0	0	0	0	0	0	0	0	0	0	0
40	0	0	1	2	2	0	0	1	1	0	0	0	0	0	0	0	0	0	1	2	0
Total	2	6	3	79	59	54	13	4	10	10	8	5	25	7	3	2	6	18	45	63	14

#### Determining weight coefficients of bottom factors

According to Eq. (2), the eigenvalues at the variable factors are the linear combination of the cumulative eigenvalues of the bottom factors and their corresponding weight values. Because the influences of the bottom factors on the corresponding variable factors and their contributions to the occurrence and evolution of accidents are different, weight coefficients were used to distinguish the contributions of the bottom factors to the corresponding variable factors. These weight coefficients could be divided into subjective and objective weight coefficients. This study adopted the improved analytic hierarchy process (IAHP) method to determine the subjective weight coefficients of the bottom factors. By optimizing the order, IAHP can self-harmoniously modify the original judgment matrix and obtain a completely consistent ordering result without any consistency checking process^23–25^. The calculation process of the weight coefficient is as follows.

1. Compare and score the relative importance of bottom pairwise factors
  The magnitude rule of Eq. (3) is adopted to evaluate the relative importance of the pair comparison of the bottom factors.
  3 $${({\mathrm{r}}_{\mathrm{i}})}_{\mathrm{jk}}=\left\{\begin{array}{l}0 \quad ({\mathrm{X}}_{\mathrm{ij}} \; \text{is} \; \text{not} \; \text{as} \; \text{important} \;\text{as} \;{\mathrm{X}}_{\mathrm{ik}})\\ 1 \quad ({\mathrm{X}}_{\mathrm{ij}} \; \text{is} \; \text{as} \;  \text{important} \;  \text{as} \; {\mathrm{X}}_{\mathrm{ik}})\\ 2 \quad ({\mathrm{X}}_{\mathrm{ij}}\;  \text{is} \; \text{more} \; \text{important} \; \text{than} \; {\mathrm{X}}_{\mathrm{ik}})\end{array}\right.$$
  where ${({\mathrm{r}}_{\mathrm{i}})}_{\mathrm{jk}}$ represents the relative importance score of the bottom factors ${\mathrm{X}}_{\mathrm{ij}}$ and ${\mathrm{X}}_{\mathrm{ik}}$; *i* is the ${i}$th variable layer factor (*i* = 1,2,3,4) ; ${\mathrm{X}}_{\mathrm{ij}}$ is the ${j}$th bottom factor under the ${i}$th variable layer factor; ${\mathrm{X}}_{\mathrm{ik}}$ is the ${k}$th bottom factor under the ${i}$th variable layer factor. The relative importance of the bottom factors is measured by the statistical frequency in Table 1. Frequency is more greater, and the relative importance is more greater. The probability that the frequency of two bottom factors is exactly the same is very small, so it is unreasonable to think that the importance of both factors is the same only when the frequency is strictly equal. Therefore, in this paper, when the difference in the frequencies of two bottom factors was not more than 10%, they were considered to have the same importance. That is, it means that ${\mathrm{X}}_{\mathrm{ij}}$ has the same importance with ${\mathrm{X}}_{\mathrm{ik}}$ as long as $\left|\frac{{{({\mathrm{m}}}_{\mathrm{i}})}_{\mathrm{j}}-{{({\mathrm{m}}}_{\mathrm{i}})}_{\mathrm{k}}}{\mathrm{min}\{{{({\mathrm{m}}}_{{\mathrm{i}})}}_{\mathrm{j}},{{({\mathrm{m}}}_{\mathrm{i}})}_{\mathrm{k}}\}}\right|\times 100{\%}\le 10{\%}$. Where ${{({\mathrm{m}}}_{\mathrm{i}})}_{\mathrm{j}}$ is the frequency of ${\mathrm{X}}_{\mathrm{ij}}$; ${{({\mathrm{m}}}_{\mathrm{i}})}_{\mathrm{k}}$ is the frequency of ${\mathrm{X}}_{\mathrm{ik}}$. ${{({\mathrm{m}}}_{\mathrm{i}})}_{\mathrm{j}}$, ${{({\mathrm{m}}}_{\mathrm{i}})}_{\mathrm{k}}$ are the "total" values in the last row of Table 3, for example, ${{({\mathrm{m}}}_{1})}_{1}=2$, ${{({\mathrm{m}}}_{1})}_{2}=6$, ${{({\mathrm{m}}}_{1})}_{3}=3$.
2. Calculate the importance ranking index and judgment matrix A_i_
  By pair comparison of the frequencies of bottom factors in the same group (i.e., calculation of ${({\mathrm{r}}_{\mathrm{i}})}_{\mathrm{jk}}$), Eq. (4) can be used to calculate the importance ranking index ${{({\mathrm{s}}}_{\mathrm{i}})}_{\mathrm{j}}$ of any bottom factors.
  4 $${{({\mathrm{s}}}_{\mathrm{i}})}_{\mathrm{j}}=\sum_{\mathrm{k}=1}^{\mathrm{p}}{{({\mathrm{r}}}_{\mathrm{i}})}_{\mathrm{jk}}$$
  The judgment matrix is calculated as shown in Eq. (5).5
where ${{({\mathrm{a}}}_{\mathrm{i}})}_{\mathrm{jk}}$ is the corresponding element in the judgment matrix A_i_; *n* is the order of judgment matrix A_i_; *p* is the number of bottom factors obtained by the decomposition of the ${i}$th variable layer factor.
3. Calculate the optimal matrix B_i_ of judgment matrix A_i_.
  Element ${{({\mathrm{b}}}_{\mathrm{i}})}_{\mathrm{jk}}$ of the optimization matrix B_i_ is calculated by Eq. (6).
  6 $${({{\text{b}}_{\text{i}}})_{{\text{jk}}}} = {\raise0.7ex\hbox{${\sqrt[{\text{p}}]{{\mathop \prod \nolimits_{{\text{q}} = 1}^{\text{p}} {{({{\text{a}}_{\text{i}}})}_{{\text{jq}}}}}}}$} \!\mathord{\left/
 {\vphantom {{\sqrt[{\text{p}}]{{\mathop \prod \nolimits_{{\text{q}} = 1}^{\text{p}} {{({{\text{a}}_{\text{i}}})}_{{\text{jq}}}}}}} {\sqrt[{\text{p}}]{{\mathop \prod \nolimits_{{\text{q}} = 1}^{\text{p}} {{({{\text{a}}_{\text{i}}})}_{{\text{kq}}}}}}}}}\right.\kern-\nulldelimiterspace}
\!\lower0.7ex\hbox{${\sqrt[{\text{p}}]{{\mathop \prod \nolimits_{{\text{q}} = 1}^{\text{p}} {{({{\text{a}}_{\text{i}}})}_{{\text{kq}}}}}}}$}}~~~\left( {{\text{q}} = 1,2, \ldots ,{\text{p}}} \right)$$
  Then, the optimization matrix ${\mathrm{B}}_{\mathrm{i}}={[{{({\mathrm{b}}}_{\mathrm{i}})}_{\mathrm{jk}}]}_{{\mathrm{p}}\times {\mathrm{p}}}$
4. Compute the weight value of the bottom factor.
  The weight value of the bottom factor ${\mathrm{X}}_{\mathrm{ij}}$ is calculated as shown in Eq. (7).
  7 $${{(\upomega }_{\mathrm{i}})}_{\mathrm{j}}={\upomega }_{\mathrm{ij}}=\sqrt[{\mathrm{p}}]{\prod_{{\mathrm{q}}=1}^{\mathrm{p}}{{({\mathrm{b}}}_{{\mathrm{i}}})}_{\mathrm{jq}}}$$
  After normalization, the normalized weight coefficient is calculated by Eq. (8).
  8 $${{(\upbeta }_{\mathrm{i}})}_{\mathrm{j}}={\upbeta }_{\mathrm{ij}}=\frac{{\upomega }_{\mathrm{ij}}}{\sum_{\mathrm{j}=1}^{\mathrm{p}}{\upomega }_{\mathrm{ij}}}$$
  According to Eqs. (3)–(8) and the data results in Table 3, the weight coefficients of the bottom factors were calculated by Matlab programming and listed in Table 4.

**Table 4:** Weight coefficients of bottom causal factors.

Bottom causal factors	Weight value	Bottom causal factors	Weight value
X_11_	0.0434	X_31_	0.0814
X_12_	0.1427	X_32_	0.434
X_13_	0.0756	X_33_	0.2634
X_14_	0.469	X_34_	0.0457
X_15_	0.2694	X_35_	0.0277
X_21_	0.5012	X_36_	0.1478
X_22_	0.1511	X_41_	0.1377
X_23_	0.0295	X_42_	0.2755
X_24_	0.1346	X_43_	0.5128
X_25_	0.1346	X_44_	0.074
X_26_	0.049

### Establishment of causal factors model

**Figure 1:**
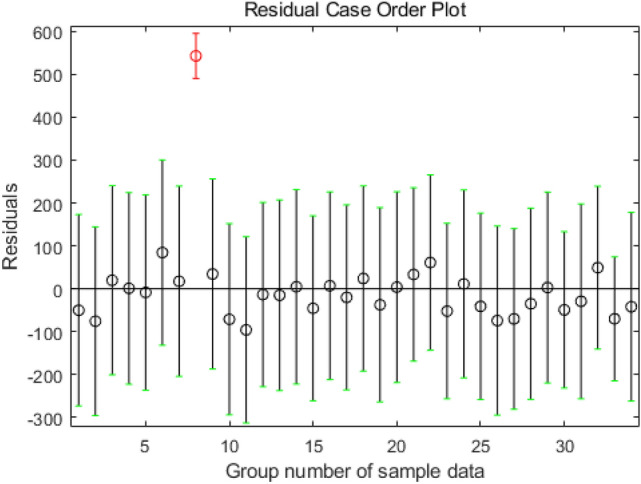
Residual analysis graph of fitting data.

According to “Modeling method of causal factors model” section, the mapping relationship between the variable layer factors and the bottom factors had been established, and eigenvalues of variable layer factors had been obtained. On this basis, the influence model between causal factors and accident consequences could be established only by determining the eigenvalue coefficient of each variable layer factor in Eq. (1) and the constant of the model. These eigenvalue coefficients and constant are actually the regression coefficient ${a}_{i}$ and characteristic constant ${a}_{0}$ in Eq. (1). Therefore, in the paper, 34 sample data sets were used as the output values, $y(j)$, and input values, ${f}_{i}(j)$, and regression coefficients ${a}_{i}$ and characteristic constant ${a}_{0}$ could be calculated using the least squares method. Through Excel and Matlab programming, the respective variables were subjected to multiple regression analyses to obtain fitted value $\widehat{y}\left(j\right)$ of dependent variable $y(j)$ and characteristic value ${f}_{i}(j)$ of the variable layer factors, as listed in Table 5. The residual error is shown in Fig. 1.

**Table 5:** Fitting value of each variable in 34 groups of test data.

Seq	Residual ($\varepsilon (j)$)	$y(j)$	$\widehat{y}(j)$	${f}_{1}(j)$	${f}_{2}(j)$	${f}_{3}(j)$	${f}_{4}(j)$
1	− 51.7157	13	64.7157	0.469	0.6358	0	0.7883
2	− 75.0268	0	75.0268	0.469	0.5012	0	0.926
3	24.9952	0	− 24.9952	0.814	0	0.434	0.2755
4	5.0252	36	30.9748	0.469	0	0	0.5128
5	− 7.1391	7	14.1391	1.4768	0.5012	0.4797	1
6	90.1081	150	59.8919	0.7818	0	0	1
7	15.4047	0	− 15.4047	0.7384	0.6653	0.5154	0.2755
8	540.1491	666	125.8509	1.4768	1.5036	0.3448	2.0256
9	32.3094	5	− 27.3094	1.5202	0.6358	0.434	0.5128
10	− 71.3479	0	71.3479	0.7384	0.6523	0	1
11	− 94.9525	2	96.9525	0.7384	0.6523	0.0277	1.3011
12	− 11.5495	21	32.5495	1.4768	0.7704	0.9137	1.3751
13	− 9.5117	19	28.5117	2.2152	0.5502	0.5818	1.5868
14	5.9277	0	− 5.9277	1.6195	0.5012	0.434	0.8264
15	− 39.5299	3	42.5299	1.2074	0.5012	0.7296	1.3011
16	− 0.1172	0	0.1172	1.6764	1.2716	0.434	0.7985
17	− 29.1725	70	99.1725	1.3501	1.7728	0.2634	1.5766
18	19.8815	0	− 19.8815	0.7384	0.5012	0	0
19	− 36.8891	0	36.8891	0.814	0.6523	0.1478	0.7245
20	1.6336	18	16.3664	1.4768	0.6523	0.0457	0.7883
21	33.6783	1	− 32.6783	2.2152	1.137	1.3948	1.2015
22	56.4111	1	− 55.4111	2.2152	1.137	0.868	0.6887
23	− 63.6412	3	66.6412	1.3501	1.6677	0	1.0996
24	7.4248	0	− 7.4248	0.469	0.5012	0	0
25	− 34.1548	14	48.1548	1.3501	0	0	1.1633
26	− 78.6989	0	78.6989	0.7384	1.0024	0	1.0256
27	− 74.1377	2	76.1377	1.4768	1.7332	0.9266	1.7143
28	− 30.9748	0	30.9748	0.469	0	0	0.5128
29	10.499	13	2.501	1.6195	0.1511	0.6095	1.0638
30	− 56.9038	33	89.9038	3.4226	2.8407	1.6468	3.0512
31	− 23.6578	8	31.6578	1.4768	0.3347	0.434	1.2015
32	54.8235	39	− 15.8235	2.5575	0.049	0	1.0638
33	− 65.2006	72	137.2006	4.8994	1.8058	0.6974	4.0536
34	− 43.9497	20	63.9497	2.8838	1.6872	0.9494	2.3267

As can be seen from Fig. 1, the residual value of the eighth data point exceeded expectations and could be regarded as an abnormal point. Thus, the eighth data point was dropped, and the regression analysis was repeated. By analogy, fitted value $\widehat{y}\left(j\right)$ of dependent variable $y(j)$ obtained by the regression analysis after discarding the four sets of data is listed in Table 6. Under a 95% confidence level, the significance test results for the regression coefficients of the respective variables are listed in Table 7, with the analysis of variance results shown in Tables 8 and 9.

**Table 6:** Fitting value of each variable after removing abnormal points from test data.

Seq	Residual ($\varepsilon (j)$)	$y(j)$	$\widehat{y}(j)$	${f}_{1}(j)$	${f}_{2}(j)$	${f}_{3}(j)$	${f}_{4}(j)$
1	13.1543	13	− 0.1543	0.469	0.6358	0	0.7883
2	− 1.9374	0	1.9374	0.469	0.5012	0	0.926
3	1.8919	0	− 1.8919	0.814	0	0.434	0.2755
4	–	–	–	–	–	–	–
5	− 2.3402	7	9.3402	1.4768	0.5012	0.4797	1
6	–	–	–	–	–	–	–
7	7.6711	0	− 7.6711	0.7384	0.6653	0.5154	0.2755
8	–	–	–	–	–	–	–
9	− 0.0057	5	5.0057	1.5202	0.6358	0.434	0.5128
10	− 4.9586	0	4.9586	0.7384	0.6523	0	1
11	− 5.4694	2	7.4694	0.7384	0.6523	0.0277	1.3011
12	14.6042	21	6.3958	1.4768	0.7704	0.9137	1.3751
13	− 3.2508	19	22.2508	2.2152	0.5502	0.5818	1.5868
14	− 9.9350	0	9.935	1.6195	0.5012	0.434	0.8264
15	− 3.1375	3	6.1375	1.2074	0.5012	0.7296	1.3011
16	− 5.7666	0	5.7666	1.6764	1.2716	0.434	0.7985
17	–	–	–	–	–	–	–
18	3.5092	0	− 3.5092	0.7384	0.5012	0	0
19	− 1.6303	0	1.6303	0.814	0.6523	0.1478	0.7245
20	6.6882	18	11.3118	1.4768	0.6523	0.0457	0.7883
21	− 5.0519	1	6.0519	2.2152	1.137	1.3948	1.2015
22	− 6.1409	1	7.1409	2.2152	1.137	0.868	0.6887
23	− 4.1729	3	7.1729	1.3501	1.6677	0	1.0996
24	6.7371	0	− 6.7371	0.469	0.5012	0	0
25	− 3.7032	14	17.7032	1.3501	0	0	1.1633
26	− 3.1131	0	3.1131	0.7384	1.0024	0	1.0256
27	− 1.6942	2	3.6942	1.4768	1.7332	0.9266	1.7143
28	− 1.0520	0	1.052	0.469	0	0	0.5128
29	0.7189	13	12.2811	1.6195	0.1511	0.6095	1.0638
30	8.1202	33	24.8798	3.4226	2.8407	1.6468	3.0512
31	− 4.7308	8	12.7308	1.4768	0.3347	0.434	1.2015
32	8.0536	39	30.9464	2.5575	0.049	0	1.0638
33	3.2505	72	68.7495	4.8994	1.8058	0.6974	4.0536
34	− 6.3088	20	26.3088	2.8838	1.6872	0.9494	2.3267

**Table 7:** Coefficients.

Model variables		Statistics				
		Unstandardized coefficients		Standardized coefficients	t	Sig.
		B	Std. error	Beta		
1	(Constant)	− 9.371	2.292	–	− 4.089	0.000
	f_1_	11.982	2.449	0.752	4.892	0.000
	f_2_	− 5.956	2.816	− 0.244	− 2.115	0.045
	f_3_	− 11.186	3.784	− 0.319	− 2.956	0.007
	f_4_	9.368	2.982	0.496	3.141	0.004

Dependent variable: y.

**Table 8:** Anova.

Model		Sum of squares	df	Mean square	F	Sig.
1	Regression	6056.774	4	1514.193	34.985	0.000^a^
	Residual	1082.026	25	43.281	–	**–**
	Total	7138.800	29	–	–	**–**

Dependent variable: y

^a^Predictors: (Constant), f_4_, f_3_, f_2_, f_1_.

**Table 9:** Model summary.

Model	R	R^2^	Revised R^2^	Standard estimate error
1	0.921^a^	0.848	0.824	6.5788332

^a^Predictors: (constant),f_4_, f_3_, f_2_, f_1_.

Therefore, according to Table 7, the multiple regression equation of the causal factors in the emergency response process can be established as shown in Eq. (9).

9 $${\mathrm{y}}\left({\mathrm{j}}\right)=-9.371+11.982{f}_{1}\left(j\right)-5.956{f}_{2}\left(j\right)-11.186{f}_{3}\left(j\right)+9.368{f}_{4}(j)$$

Tables 8 and 9 show that the regression equation has a good fitting effect and is representative, and the linear relationships between the regression coefficients and regression variables are significant.

### Generalization test of causal factors model

In order to verify the generalization ability of the established causal factors model, the last six groups of sample data (2015–2020) were used for predictions. Considering the strong randomness of accident system, incompleteness of statistical data and less sample data, and focusing on medium and short term data prediction, so the grey prediction method with strong versatility was selected^26^. Firstly, the GM(1,1) grey prediction method was used to calculate the eigenvalue of each variable layer in each group of data, and these eigenvalues were used as the input data of the established regression model and substituted into Eq. (3) to predict the number of possible deaths in each group of data. The prediction results are listed in Table 10. Moreover, the predicted values calculated by the regression model of the causal factors established in this study were compared with the values predicted by the GM(1,1) model alone, as listed in Table 11. It can be seen that the average data prediction error of the established model was smaller than that of the direct prediction by GM(1,1), and the prediction effect was better than that of the GM(1,1) model alone.

**Table 10:** Accident prediction data for 2015–2020.

Year	Relative error	Residual	Actual value	Prediction value	F_1_	F_2_	F_3_	F_4_
2015	12.4%	1.12	9	7.88	0.7384	2.7917	0.4340	3.1890
2016	–	− 0.04	0	0.04	0.3446	1.3132	0.1440	1.5706
2017	− 6.0%	− 0.30	5	5.30	0.4801	0.6294	0.1121	1.4863
2018	− 0.6%	− 0.05	9	9.05	0.6690	0.3017	0.0873	1.4064
2019	2.8%	0.36	13	12.64	0.9322	0.1446	0.0680	1.3309
2020	5.6%	1.01	18	16.99	1.2990	0.0693	0.0530	1.2594

**Table 11:** Comparison of accident death toll prediction effects.

Year	Actual value	Established model prediction	Absolute residual	Relative error absolute value	GM (1,1) model prediction	Absolute residual	Relative error absolute value
2015	9	7.88	1.12	12.4%	9	–	–
2016	0	0.04	0.04	–	3.54	3.54	–
2017	5	5.30	0.30	6.0%	5.56	0.56	11.2%
2018	9	9.05	0.05	0.6%	8.75	0.25	2.8%
2019	13	12.64	0.36	2.8%	13.76	0.76	5.9%
2020	18	16.99	1.01	5.6%	21.64	3.64	20.22%
Mean error	–	–	0.48	5.48%	–	1.75	10.03%

### Model error analysis

The main factors affecting the accuracy of the established causal factor model for emergency responses in this study were the randomness and uncertainty of the accident itself. The randomness of accidents was manifested in the fact that the causal factors of accidents appeared randomly, and the evolution of an accident was affected by the interaction of the internal causal factors of the accident system and the random influence of the external environment. This made the relationships between accident consequences and causal factors more uncertain. The main sources of errors in the model constructed in this study included the following three aspects.

1. Sample size. This study collected and sorted fire and explosion accident cases involving typical oil depots at home and abroad over the past 50 years by conducting a literature review, consulting online public reports, and performing enterprise research. Because of data acquisition limitations, there may be incomplete statistics on accident cases.
2. Sample reliability. Because of the incomplete disclosure of some accident case data, there may be incomplete data and uncertain reliability in the accident cases investigated in this study. This may have affected the accuracy of the eigenvalues in the bottom factors, thereby affecting the accuracy of the model.
3. Weight coefficients. The weight distribution of the bottom factors of the model was one of the important factors affecting the accuracy of the model. A more reasonable weight distribution could make the model more inclined to the objective law of the accident evolution process. However, because the relationship between the evolution of accidents and the causal factors has not yet been accurately described, the subjective weighting method was selected to determine the weights, which introduced errors in the calculation of the weights.

Therefore, in order to improve the accuracy and generalization ability of the model, statistical methods (such as an analysis of variance and cross validation) could be further considered to reduce the randomness and uncertainty between the causal factors and the consequences of the accident, thereby reducing the error of the model.

## Causal factor evaluation and corresponding preventive measures

### Causal factor evaluation based on mutation progression method

Effective accident prevention countermeasures can be formulated only when hidden dangers are found from the causal factors. Therefore, it was necessary to evaluate the relative contributions of the causal factors and determine their changing trends so as to provide more targeted and prioritized prevention measures for the causal factors. There are many methods to evaluate the relative contributions of causal factors, such as the fuzzy evaluation method, analytic hierarchy process, mutation progression method, and expert evaluation method. Among these, the mutation progression method does not need to consider the index weight, but considers the relative importance of each evaluation index, overcomes the subjectivity in the weight distribution process, and is suitable for solving multi-objective decision-making problems^27^. Therefore, the mutation progression method was used to evaluate the relative contributions of the causal factors in this study. There were four variables in the regression model of the causal factors in this study. Therefore, the butterfly mutation model was used to evaluate the causal factors. The normalization formula of the butterfly mutation model is shown in Eq. (10):

10 $$\left\{\begin{array}{c}{X}_{{F}_{1}}=\sqrt{{F}_{1}}\\ {X}_{{F}_{2}}=\sqrt[3]{{F}_{2}}\\ {X}_{{F}_{3}}=\sqrt[4]{{F}_{3}}\\ {X}_{{F}_{4}}=\sqrt[5]{{F}_{4}}\end{array}\right.$$

where ${F}_{1}$, ${F}_{2}$, ${F}_{3}$, and ${F}_{4}$ are the variable layer factors; and ${x}_{{F}_{i}}$ is the evaluation value of the mutation progression of the ${i}$th variable layer factor.

The sum of the evaluation values of the mutation progression of each causal factor in the variable layer is the relative dangerous state parameter ${D}_{h}$, as shown in Eq. (11).

11 $${D}_{h}={x}_{{F}_{1}}+{x}_{{F}_{2}}+{x}_{{F}_{3}}+{x}_{{F}_{4}}$$

**Figure 2:**
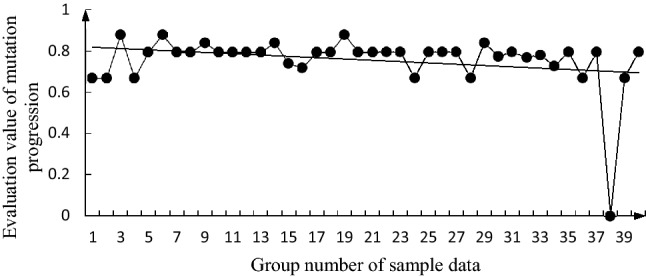
Change trend graph for ${x}_{{F}_{1}}$.

**Figure 3:**
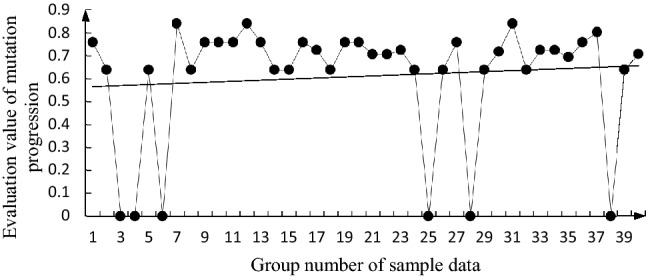
Change trend graph for ${x}_{{F}_{2}}$.

**Figure 4:**
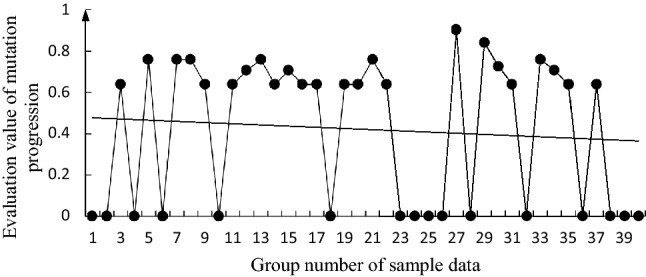
Change trend graph for ${x}_{{F}_{3}}$.

**Figure 5:**
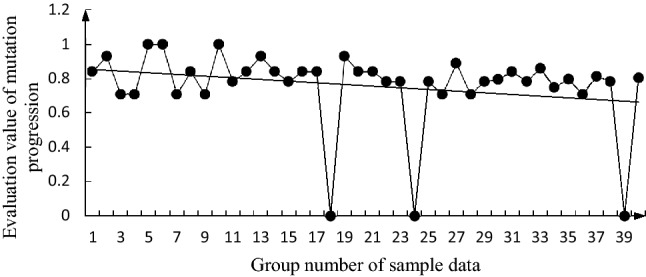
Change trend graph for ${x}_{{F}_{4}}$.

**Figure 6:**
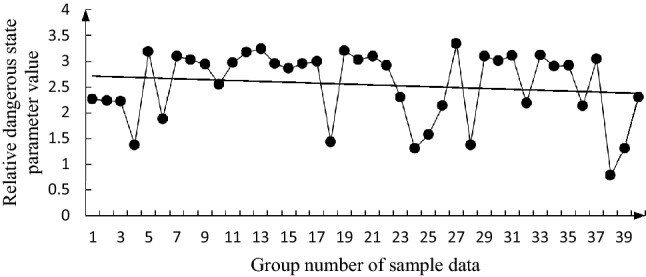
Change trend graph for ${D}_{h}$.

The specific calculation steps for the butterfly mutation are detailed in the literature^28^. Taking the annual cumulative value of the bottom factors in the 40 groups of sample data as the measured value of the evaluation factor, according to Eqs. (10) and (11), and the data in Table 3, the evaluation values (${x}_{{F}_{i}}$) of the mutation progression of each group were calculated. The change trends of these evaluation values are shown in Figs. 2, 3, 4, 5, 6.

It can be seen from these figures that ${x}_{{F}_{1}}$, ${x}_{{F}_{4}}$, and ${x}_{{F}_{3}}$ generally show a downward trend; ${x}_{{F}_{2}}$ has a deteriorating trend; and ${D}_{h}$ has a general downward trend. The changing trends of ${x}_{{F}_{1}}$, ${x}_{{F}_{4}}$, and ${x}_{{F}_{3}}$ are similar to those of ${D}_{h}$. Although ${x}_{{F}_{1}}$, ${x}_{{F}_{4}}$, and ${x}_{{F}_{3}}$ can be considered to be the main factors affecting ${D}_{h}$, ${x}_{{F}_{2}}$ has a deteriorating trend and needs to be considered. From the 39 and 40 sets of data, ${x}_{{F}_{1}}$,${x}_{{F}_{2}}$,${x}_{{F}_{4}}$, and ${D}_{h}$ all show a deteriorating trend, which indicates that the fire safety situation is not optimistic, and the consequences of a secondary accident during oil storage system may rebound.

### Analysis of preventive measures

People's professional skills, knowledge and literacy, environmental issues, and firefighting facilities are the main factors leading to secondary accidents in oil storage system. These three types of factors generally show a trend of gradual improvement. However, according to the last two sets of data (groups 39 and 40), the mutation progression values of people's professional skills, knowledge, and literacy show an upward trend, which requires close attention. Inherent objects or equipment are generally deteriorating, but according to the last two sets of data (groups 39 and 40), their mutation progression values have dropped significantly. Therefore, from a macro point of view, inherent objects or equipment are the focus of prevention, but the other three types of factors have not completely improved and cannot be ignored. Therefore, based on the evaluation results for each influencing factor, the following preventive measures are proposed.

1. Inherent objects or equipment: Strengthen regular inspections of tanks, valves, and other inherent facilities, with the timely elimination of potential safety hazards such as corrosion and cracks in oil tanks. Closely monitor whether the tank body stress and settlement deformation exceed the safety requirements. Regularly inspect oil depots to prevent oil leakage.
2. People's professional skills, knowledge, and accomplishments: Strengthen fire safety training for employees and ensure that they are familiar with operating procedures and precautions. Enhance awareness of safety laws, enact strict management mechanisms, and prevent illegal operations.
3. Environmental aspects: Design the structures and layouts of factory buildings in accordance with relevant national regulations, and ensure fire separation between buildings in a reservoir area.
4. Firefighting facilities: It is necessary to perform regular maintenance, inspections, and renewal work to ensure that these can be put into use at any time to prevent long-term disrepair and failure. Carry out practice exercises to ensure that employees are skilled and conduct appropriate operations.

## Conclusion

This study focused on emergency safety by modeling and evaluating the causal factors in the emergency responses to fire accidents involving oil storage system. Consequently, the following conclusions can be drawn from the research reported in this paper.

1. Based on the principle of multi-factor hierarchical modeling, the causal factors in the emergency response processes were decomposed into criterion, variable, and bottom layers. The corresponding relationships between the bottom layer factors and variable layer factors were established using the mapping rule, and a multiple linear regression model between the variable layer factors and accident consequences was established using the regression analysis method. This made it possible to finally establish a quantitative model between the causal factors in the emergency response processes and the accident consequences.
2. By combining the established regression model of the causal factors in the emergency response processes with the GM(1,1) grey prediction method, the severity of accident consequences was predicted, and it was found that the prediction result was more accurate than that when using the GM(1,1) model alone and more in line with the actual accident results.
3. The mutation progression evaluation values for the causal factors and their change trends were calculated using the butterfly mutation model. The relative importance and degree of influence of the causal factors on the accident consequences were obtained, and targeted preventive countermeasures were proposed.
4. Through the establishment of a quantitative model between the causal factors in the emergency response processes and the consequences of fire accidents involving oil storage system, and the quantitative evaluation of the degrees of influence of the causal factors on the accident consequences, this study provided an important theoretical basis for revealing the occurrence and evolution mechanism of secondary accidents during emergency response processes and realizing proactive protection beforehand.
